# Extra Supplement—The Nursing Mirror

**Published:** 1890-04-05

**Authors:** 


Being the Extra Nursing Supplement of "The Hospital" Newspaper.

All Contributions for this Supplement should be addressed to the Editor, The Hospital, 140, Strand, London, W.C., and should have the word "Nursing" plainly written in left-hand top corner of the envelope.

### En Passant.

**Bolton Nursing Association.**—The first annual meeting of this Association was satisfactory, no fewer than 303 cases having been attended, and the staff has had to be increased. Both Lincoln and Bolton tell the sad tale of a nurse contracting fatal sickness in the discharge of her duties. Miss Yale has lately been appointed matron at Bolton; she trained at Bloomsbury.

**Lincoln Institution.**—The annual report tells of increased work and satisfactory funds. There are now seven nurses belonging to this institution working amongst the poor of Lincoln. The amount of bonus allowed on the nurses' investments has had to be reduced. Why not join the Pension Fund at once, and let the nurses share in the great generosity of the public towards them?

**Loyalty.**—We all know how boys brought up at a public school ever believe that school the best in all England, and struggle both in examinations and athletics to maintain its supremacy amongst all comers. We should like to see more of this spirit amongst nurses, especially as the training schools are beginning to do far more for their pupils than entered into the contracts of a few years ago. But this spirit of loyalty would be utterly crushed by a common register such as the B.N.A. propose to establish; more especially as the Nurse Training Schools have declared against the register, so that to persuade nurses to join it, is to put them in direct opposition to their old school and teachers.

**Short Items.**—Lady Dufferin, ever interested in nursing, lately visited St. Paul's Home for Nurses at Rome.—Dr. Perry, the warden of the new college at Guy's, is engaged to be married to Miss McMasters, late Sister at the London Hospital.—The district nurse at Penzance is at home two afternoons a week to attend to patients who call, and who pay 2d. for her ministration. Is not this rather likely to tempt the nurse to prescribe, and to go beyond her province?—Prize giving took place last week at the two Edinburgh Schools of Medicine for Women. The ladies passed well at the late examinations.—A nurse lately asked a physician how she should proceed to become a masseuse. He replied: "Study anatomy and physiology at one of our medical schools, and then go to Stockholm for at least three years' thorough instruction."

**Thousands of Pounds for the National Pension Fund.**—We have the pleasure to acknowledge the following sums towards the £14,000 now being raised to at once bring the Donation Bonus Fund up to £40,000. We hope to be able to acknowledge contributions amounting to £1,000 every week until the £40,000 are completed. Contributions of any amount may be sent to the Editor of The Hospital, or to the Manager, National Pension Fund for Nurses, 8, King Street, Cheapside, London, E.C.:

Eleventh Thousand.

| | £ | s. | d. | | £ | s. | d. |
|---|---|---|---|---|---|---|---|
| Messrs. Baring Brothers and Co. ............ | £500 | 0 | 0 | Messrs. W. T. Carr, Sons, and Tod ............ | £21 | 0 | 0 |
| Messrs. Cox & Co. ......... | 200 | 0 | 0 | Messrs. Robarts, Lubbock & Co. ......... | 21 | 0 | 0 |
| Mr. J. Travers Smith ... | 50 | 0 | 0 | Duke of Northumberland ............ | 10 | 10 | 0 |
| Messrs. Markby, Stewart & Co. ............ | 50 | 0 | 0 | Mr. C. A. Ionides ......... | 10 | 10 | 0 |
| Exors. of Mr. D. Proctor | 50 | 0 | 0 | Mr. J. A. de Lacheur ... | 10 | 0 | C |
| Mr. W. R. Sandbach ... | 50 | 0 | 0 | Mr. J. Barlow ........... | 0 | 10 | 0 |
| Mr. Steinkopff ............ | 25 | 0 | 0 | Small accounts ............ | 0 | 15 | 9 |

Amount already received Eleven Thousand Pounds.

**District Nursing at Staleybridge.**—For the past five years there have been two district nurses at Staleybridge, who have faithfully fulfilled the duties of sick and monthly nurse. During the past year there was a great demand for a night nurse, as it was impossible for the nurses to watch by their patients at night, as well as in the daytime. Finally, a nurse has been summoned, and is filling with patience and perseverance the post of night nurse. It is to be hoped that the district nursing under the thoughtful supervision of Mrs. T. F. Knott, Mrs. T. B. Hall, and several others, will, in the future, prosper and be as much comfort and aid to those in sickness and suffering as it has always proved itself in the past to be.

**Exhibition of Nursing Uniforms.** — The dressers of the nurse-dolls will be glad to hear that they are once more safely in town, and will shortly be on view at our office. The dolls have practically benefited Charing Cross Hospital to the extent of £10, and the Liverpool Hahnemann Hospital and the Salop Infirmary to the extent of £3 each. They have also earned a small sum for the National Pension Fund for Nurses. But this is only a part of the work of the doll-nurses; they have raised a considerable interest in the question of uniforms, and they will ever be of the greatest assistance to matrons who desire to choose a new garb for their nurses. They have also interested many people in nursing who before thought little of the subject. We are sure all those nurses who spent so much time over the dolls, will feel recompensed for their labours by the success with which the dolls' tour has been attended.

**Nursing in Chicago.**—The nurses of St. Luke's Hospital, Chicago, train for two years, and receive excellent lectures from the medical staff, and weekly classes held by the superintendent (Miss K. L. Lett). Nurses are on duty from eight a.m. to eight p.m., with an occasional two hours off duty; they are supplied with a uniform and a small salary to cover expenses, but in nowise intended as wages. There are twenty-three nurses in the school, which number must shortly be increased to thirty-eight to provide for the nursing of the Johnston Memorial Hospital. The demand for private nurses exceeds the supply. The past and present nurses have lately banded themselves together to form a "Blue Cross Association" to help one another in time of sickness. Dr. Samuel J. Jones, in addressing the last graduating class, spoke of the rise of the present scientific nursing, and said: "This coming light first dawned in the old world where noble women, by their good work and excellent example, showed that nursing is not menial employment, but an honourable vocation, when educated heads and deft hands unite with benevolent feelings to assuage the sufferings of humanity. The work of the nurse then becomes intelligent aid to the physician, just as the physician's work is but an aid to nature in her kindly efforts to repair the damage done by violation of her laws, as shown in what we are accustomed to call disease—which is but another name for the penalty that must always be paid for violating those laws. America soon saw and appreciated the good work of those devoted women, whose intellectual and social advantages well fitted them to undertake a reform so much needed, and the contagiousness of good work—like that of evil-doing—soon became manifest on both sides of the Atlantic, and spread from the seaboard to the interior of our country. Through the commendable foresight of our public-spirited women and generous men, we have had provided the schools which afford the needed facilities for educating nurses, that are just as useful and just as honourable, in their way, as are the medical schools, designed to educate physicians."

### Nursing Lectures.

By Percy Lewis, M.D.

Lecture VII.—ABDOMINAL DISEASE.

The special symptoms to be noted in abdominal diseases are the appetite, vomiting, pain, flatulence, hiccup, and the motions.

The appetite is affected more or less in most diseases, and is a symptom which it is always important to note. You should get into the habit of estimating by the eye how much by weight a patient takes. For instance, in a report you would not put down "appetite a little better" or "a little worse," but about how many ounces of the different articles of food the patient had taken. Notice also at what times he feels most inclined for feeding. In hospitals it is especially necessary to keep a record of whether patients eat their food or not, for when they leave they frequently make complaints against the food, which complaint gets taken up by some well-meaning person, and which it is very difficult to disprove unless it is all recorded. When vomiting occurs it should be noted how soon after food it takes place, whether preceded or followed by pain, whether the food is digested or not, whether frothy or any special colour; also if it is brought on by anything special, such as syringing an ear, painting the throat, any special article of diet, or by any special position, as lying on the left side or on first rising in the morning. Vomiting, in fact, depends on a variety of causes, in the finding out of which an intelligent nurse may render us great assistance. Vomit should always be kept for a doctor to see, and the quantity measured. The stomach may get blocked at the further end, and gradually dilated. In this condition the food may accumulate for days until it amounts to several quarts, when it gets vomited. If the intestines get obstructed so that nothing can pass at all, the contents of the bowel are regurgitated into the stomach, and fæcal vomiting results. If the food is fermented in the stomach a frothy vomit is formed. In some diseases, as gastric ulcer, or gastric congestion in heart or liver disease, blood is poured into the stomach and vomited. When in large quantity it is brought up quickly, its nature being obvious; but when in smaller quantities it becomes altered by the gastric juice, and from being a bright red it is changed to a dark brown or black. In small quantities it is described as coffee grounds. Vomiting of blood is called hœmatemesis.

Pain may be associated with vomiting, as happens when a stone passes from the gall bladder or the kidney. Sometimes pain is relieved by pressure, but at others the abdomen is so tender that even the weight of the bed clothes cannot be borne. This is the case in peritonitis, when it is necessary to have a cradle to keep off the weight of the bed clothes. Colic is a painful spasmodic contraction of the intestine, brought on chiefly by indigestible food. Pain in the abdomen may be due also to many other conditions, as abscess, tumours, aneurisms, &c., which I mention to impress on you the importance of noting and accurately reporting. In a hospital always send for the house surgeon if a patient is in pain. No house surgeon will object to come to a patient in pain at any time; still, use your judgment, and don't send for him every time an old woman gets the wind.

Hiccup is only of importance when prolonged, as it may be in obstruction of the bowels.

With regard to the motions, you must become well acquainted with the different special characters associated with different diseases. As a rule, you are not expected to give your opinion as to whether they are typhoid or dysenteric, &c., but to describe them according to their colour and consistence. You must notice whether they are formed and of natural size, watery, or worm-like, which happens in some cases of obstruction. In colour they may be black, green, clay colour, pea-soup colour, &c. When black and of tarry consistence there is probably a good deal of blood, altered by the intestinal secretions, mixed with them. Should the blood be much in quantity and unaltered, it has been poured out into the intestine quickly, and is probably still being poured out. In this case send at once for a doctor, and in the meantime keep the patient as quiet as possible. Various medicines, such as iron, bismuth, and charcoal, also produce this black effect. Sloughs have to be looked for in typhoid; they are difficult to describe, but once seen they will always be recognized again.

The medical diseases of the liver are not very interesting from a nursing point of view. It may become enlarged in heart disease, or contracted, "cirrhosed," from excessive use of alcohol. In the latter case it is often associated with dropsy, or bleeding from the nose. Jaundice is produced from retention of bile in the blood, generally from some obstruction in the bile ducts. The obstruction may be due to different causes, as catarrh or swelling from chill, tumours, and wedging of gall stones..

Abscesses of the liver are common in tropical countries, but you will frequently see cases here in sailors who come from India to be cured. If not operated on, these abscesses may open on the abdominal wall, into the stomach, when the pus will be vomited, or into the lungs, when it will be coughed up.

Hepatic colic is the pain produced by the passage of a gall stone down the duct which connects the liver with the duodenum. The pain is at times simply agonizing, the features becoming pinched and expressive of great suffering, the skin cold and covered with perspiration; vomiting commonly accompanies it. As a rule the pain is not continuous, but recurs again and again until the stone passes into the intestine.

The most important part of the nursing of abdominal cases is included under the head of feeding, which forms the subject of the next lecture. I will now finish with a reference to three minor points. Mustard is used for purposes of counter irritation, as poultices mixed, or not, with linseed, or as the ready-made preparation called mustard leaves, in which form it is mostly ordered. These are moistened in tepid water, covered with cotton wool, and fastened with a bandage. They should not be left on more than half an hour, and after removal should be replaced by cotton wool. Fomentations should be changed every half hour at least—oftener if you have only one case to attend to. If turpentine is ordered, about thirty or forty minims is the right quantity to sprinkle on them. Fomentations should be covered with mackintosh or jaconet, which should everywhere overlap the flannel. In using spongiopiline for fomentations or liniments, remember that it can be used a great number of times, and isvery expensive.

### Presentations.

Miss F. E. Cole, lately appointed matron of the Dartmouth Cottage Hospital, has been presented with a beautiful seal skin travelling bag, a purse of sovereigns, and address, upon her leaving Rickmansworth, where she has ministered to the sick and suffering with such devotion and unselfishness during a period of five years. The Rev. A. E. Northey, vicar, who made the presentation in the name of the subscribers, who mostly assembled at the vicarage to wish her happiness, and health in her new sphere of work.

Mr. F. C. Knight, Clerk to the Liverpool City Hospitals, has been presented with a watch on the occasion of his resignation. The presentation was made by Dr. Bond, at the City Hospital in the presence of many of the officials and nurses, and a pleasant evening was spent.

### News from Tasmania.

Does Tasmania seem to be so far off and so strange as to be without interest to English nurses? Surely not, for many have emigrated here and many more are likely to, as this lovely little island becomes better known as a health resort. The Hospital penetrates to us here, and in return for the interesting news of our comrades which we gather from its pages, we send you an account of our medical charities. This is a most beautiful country, and the climate is delightful. The rivers are like arms of the sea with large hills on either side, and the whole place is luxuriant with tree ferns. But to turn from the country to the good works done therein and commence with the chief hospital.

Hobart General Hospital.—Matron, Miss Harriet Munro, trained at St. Bartholomew's and Children's Hospital, Pendlebury. Number of beds, 80 for male general cases, 28 for women general cases, and 28 fever beds. Five sisters (one on night duty) in charge of the whole hospital. Each sister, except the sisters of the fever ward, takes night superintendence in turn for three months. The staff varies from eighteen in number at slack times, to twenty-five in busy fever seasons. All ward work is done by the ward maids, two of whom work on each floor. Probationers come on two months' trial, at the end of which time they enter into a contract to serve for two years; at the end of two years they are examined by the lady superintendent, and by the senior medical officer, and if found proficient a printed certificate is given. The head nurses or sisters were formerly ladies who had come from English hospitals, but now every sister in the hospital has been trained in Hobart Hospital alone, the last remaining who had been trained elsewhere having been recently appointed to the matronship of Melbourne Hospital. Miss Rathie is appointed to the Melbourne Hospital; she came to the Colony some years ago from the Edinburgh Infirmary, where she received her training. Her work at Melbourne will be extremely difficult; we wish her success. Miss Harriet Munro, now matron of Hobart Hospital, was, when she first came out, matron of the Nurses' Home, Philip Street, Sydney. As I said once before, ladies' experience there is rough. Over-skilled nurses are not required so much as nurses who will help in the general work of the houses they have to go to. Miss Munro testified that sometimes they are required to cook as well as clean firesides, &c. Hence much trial and disappointment to lady nurses fully trained who come out from England. Miss Munro has not been able to persuade the doctors in Hobart to give lectures to the nurses. She has supplemented that want by giving some herself. No nurses in Hobart Hospital are under twenty years of age, twenty-five years of age is preferred, and the sisters are usually over twenty-six years old. Printed certificates given to the nurses are almost the same as those given at St. Bartholomew's Hospital, London. In the Hobart Hospital there are eight ward maids. The nurses do no ward work, except dusting and cleaning medical or surgical apparatus. The hospital is very pleasantly situated in a good street, with views at the back of the beautiful Hobart Bay and the hills on the other side of the water. There are verandahs and balconies, with glass shutters that can be closed or open at pleasure, which are very popular among the patients and extremely useful to them. Part of the hospital was an old priory, and part is newly built. The former is more convenient than the latter. At present the nurses live in an old house across the street—a new one is to be built shortly.

Cascades Hospital for Insane, Hobart.—This institution is more immediately set apart for male criminal lunatics, the majority of whom were removed from Port Arthur establishment in April, 1877. The average number of daily inmates in 1887 was 57, and the cost per head £39 18s. 4d. The expenditure increased during the year by the introduction of gas throughout the building, which amounted to £2,302, of which £1,077 was chargeable to the Imperial Government. There was no nursing in the right sense of the word in this establishment, and several abuses that were overlooked by a doctor aged 80 have been rectified. The Dean of Hobart has succeeded in getting some improvements in the management of the inmates, at, of course, considerable ill-will towards himself. There were innumerable letters in the newspapers against any reform, some even from clergymen, who ought to have known better. The depôt at Hobart is at New Town, and is a sort of benevolent asylum. It is in a fine and healthy situation. There are generally over 100 in hospital bedridden. The nursing is done, as it happens, among the patients themselves. There is accommodation for 500 males and 200 females. The Matron is Louise Hurst; Nurse, Catherine Robinson. Expenditure, £8,587 0s. 5d.

Boys' Training School Reformatory is at the Cascades attached to the Lunatic Asylum. The boys are instructed in agriculture, gardening, carpentering, with a view to licence at proper time to suitable employment. Mark system. Matron, Mrs. Farquhar. Accommodation for 50.

The Hospital for Insane is at New Norfolk. Matron, Miss S. Alexander; Sub-Matron, Miss J. R. Ayres. Average number in 1887, 288. Total treated, 340. Gross cost of institution, £10,821 3s. 7d.

Central Committee for Boarding out Destitute Children at Different Places.—In 1888 the total benefited was 100.

Launceston General Hospital is conducted much on the same lines as that in Hobart. Lady superintendent, Miss Milne, one of three sisters who came out from the Edinburgh Infirmary; number of beds, 94; nurses, 13; average number of in-patients daily for 1887, 73¾; expenses, £5,224 15s. 3d.; net cost of each bed occupied for the year, £51 10s. 3d.; in-patients, 940 in 1887; out-patients, 594.

Launceston Invalid Depôt for Males and Females.—Average number of inmates, 148; cost, £2,050 5s. 11½d.; per head, £13 7s. 2d.

### Everybody's Opinion.

*[Correspondence on all subjects is invited, but we cannot in any way be responsible for the opinions expressed by our correspondents. No communications can be entertained if the name and address of the correspondent is not given, or unless one side of the paper only be written on.]*

### MEUM AND TUUM.

Periwinkle writes:—I am in sympathy with "Justine's" bold and spirited attack upon existing though rarely acknowledged evils among us, and am far from agreeing with "Handclasp" that it is like putting "the cart before the horse" for a complaint against "the nurse" to be made by one of ourselves—in other words, perceiving the beam in our own eye as well as the mote in our brother's. Small chance, indeed, is there of making our profession a higher calling than it is unless we do ourselves clearly recognise, honestly acknowledge, and patiently strive to root out every evil that besets it.

### A PROTEST FROM THE RANKS.

Miss E. Foster writes:—I have taken in your valuable paper, The Hospital, for some time past, and derived both pleasure and profit from its pages, but in The Hospital for the week ending March 22nd I observed an article about the Society for the Prevention of Cruelty to Animals, in which the prevailing idea seems to be that only women who dwell in drawing-rooms have any sympathy with suffering (I may have misunderstood it certainly), but, if this is so, I feel it to be incumbent on me to correct such a mistake. I do not reside in a drawing-room myself, having to work very hard for my living, but I have just as much sympathy with the dumb and helpless as if I did—perhaps more so—and being possessed of a highly-strung nervous organization, cruelty to anything affects me quite as much as it would my more highly favoured sisters, more especially when I feel my own inability to prevent such cruelty. I have no doubt there are many more who feel the same about these things, if one did but know them, who yet belong to the masses, and not what is called the classes.

### NURSES' MUTUAL SOCIETY.

N. E. F. writes:—Having heard that the nurses are on the eve of starting a mutual society, I should like to wish them every success in their undertaking. At the same time, if I were a nurse I should be chary of connecting myself with any society until it was firmly established. This is an age of societies and trades unions, but how few of them succeed. One of the reasons of this is, I think, that the members are not always up to the highest standard in their work. Now I do trust that in this institution only nurses thoroughly trained in hospitals will be admitted, and that each nurse may only be sent to cases of which she has previous knowledge and practice. This is often not so, as frequently inefficient persons are sent out, as I know by experience, having had during the last few years much to do with nurses. The general public has a much better knowledge of nursing in the present day than in former times, and nurses are not now sent for unless patients are in a really precarious condition, or in cases where surgical and technical knowledge is required. It is, therefore, most annoying to have an attendant sent who has very little more skill than oneself. I trust that the lady superintendent is a good disciplinarian and manager. Many nurses will do well with some one over them, but take away the guiding power and they are of very little use. On the other hand, some work better on their own responsibility, and will brook no interference.

### Examination Questions.

### PRIZE ANSWER FOR MARCH.

THE principal divisions of fever are epidemic, endemic, and sporadic. *Epidemic* means a disease which is infectious from one person to another and which goes from place to place, the fever germs being reproduced in the human body; as small-pox, scarlatina, influenza, measles, &c. A fever is called *endemic*, when the disease occurs only in certain districts, and is confined to that part; for instance, we find intermittent fever (ague) in cold damp places—whilst remittent fever is almost entirely confined to the tropics. The term *sporadic* means scattered; it is applied to those cases which occur severally, or in scattered numbers. Fevers are also divided into *Idiopathic*, and *Symptomatic*. In the former the fever is the principal feature of the disease; e.g. scarlatina, whilst in the latter division it is merely one symptom amongst the rest. The fever in pneumonia is called *symptomatic*. *Eruptive* fevers are those in which the appearance of the eruption, or rash, is the climax of the disease—as in measles and chicken-pox. In *continued* fevers, the rash does not seem to have any particular effect, the temperature continuing high, in spite of it; typhus and typhoid are continued fevers. The word *zymotic* as applied to fevers, signifies those diseases which are preventible. Small-pox comes under this heading, also *typhus*; the result of over crowded houses, and insufficient ventilation; and *typhoid* fever, which arises from bad drainage and impure water.

### Notes and Queries.

TO CORRESPONDENTS.—1. Questions may be written on post-cards. 2 Advertisements in disguise are inadmissible. 3. In answering a query please quote the number. 4. If a private answer is desired, a stamped addressed envelope must be forwarded.

NOTICE.—We must really ask our correspondents to be more particular in enclosing name and address. Constantly we receive queries or letters with no name attached.

### Queries.

(72) *Food.*—What is considered the most blood-forming food that can be taken?—*M. W.*

(73) *Pharmacy.*—Where and how can a nurse be instructed in pharmacy, and what is the probable cost?—*Philada.*

(74) *Mental Nursing.*—Where can I receive training as a mental nurse?—*F. J.*

(75) *Medical Dictionary.*—Kindly tell me the name and price of a cheap medical dictionary.—*L. D.*

(76) *Caps Wanted.*—Will anyone kindly give me a pattern of, or tell me where I can procure, nurse's caps, tying under the chin?—*Capless.*

### Answers.

(75) *Medical Dictionary.*—"Hoblyn's Dictionary," price 10s. 6d., published by Whittaker and Co.

*Ex-Matron.*—Do you desire your last letter published? If so, please re-write it on one side of the paper only, and send it us again.

*M. E. W.*—The chief out-door uniforms were described in THE HOSPITAL for January 11th.

### Appointments.

[It is requested that successful candidates will send a copy of their applications and testimonials, with date of election, to THE EDITOR, The Lodge, Porchester Square, W.]

CROYDON GENERAL HOSPITAL.—Miss Janet E. Lambton, for five years matron of Rochdale Infirmary, has been appointed matron at Croydon, *vice* Miss Elizabeth Boucher, who is retiring after 20 years' service. Miss Lambton received her training at the Albert Edward Infirmary, at Wigan, and was for two years charge nurse at Salford Royal Hospital. We wish her every success at Croydon, where, we are sure, she will receive a hearty welcome.

### Vacancies.

THE following vacancies are announced:

### Matrons.

| | |
|---|---|
| Boscombe Hospital; £35. | Fulham Union Infirmary; £80. |
| Fleming Memorial Hospital; £50. | Hanwell District School; £40. |

### Nurses.

| | |
|---|---|
| Aimee Nursing Homes. | Her Majesty's Hospital; £25. |
| Bedfordshire Institution; £27. | Home for Incurables, W. |
| Birkenhead Borough Hospital. | Hull Nurses' Home. |
| Blackheath Institution. | Jessop Hospital for Women, Sheffield (night); £25. |
| Bolton Nursing Association; £30. | Kent Institution; £30. |
| Bournemouth Institution; £25. | Kingston Nurses' Home, Hull. |
| Bridgwater Infirmary; £23. | Liverpool Eye Infirmary; £25. |
| Brompton Cancer Hospital; £25. | Lowestoft Hospital; £20. |
| Burton-on-Trent Institution; £25. | Massachusetts; £40. |
| Canterbury Hospital; £22. | Mr. Wilson's Institution. |
| Cardiff Infirmary; £24. | Pendlebury Hospital; £20. |
| Coventry and Warwickshire Hospital; £25. | Royal Chest Hospital; £22. |
| Christ's Hospital, Hertford. | Stamford Infirmary; £25. |
| Eastbourne Sanatorium; £35. | St. Helen's Cottage Hospital; £20. |
| Edinburgh Children's Hospital. | Stockport Infirmary. |
| Fitzroy House. | St. Vanda's Home, Bromley. |
| General Nursing Institute. | Western General Dispensary; £25. |
| Glasgow Victoria Hospital (night). | West Kirby Nurses' Home. |
| Guildford R.S.C. Hospital; £20. | Wonford House Hospital, Exeter. |
| Harrow Cottage Hospital; £30. | |

### District Nurses.

| | |
|---|---|
| Burton-on-Trent Institution. | Kensington Nursing Association. |
| Cardiff; £30. | Pembury; £50. |
| Gainsborough Association. | Tunbridge Wells; £50, |
| Haslemere. | Warwick. |

### Probationers.

| | |
|---|---|
| Burton-on-Trent Infirmary. | Lowestoft Hospital. |
| Burton-on-Trent Institution. | Nottingham Nursing Association. |
| Chalmer's Hospital, Banff. | Nurses' Home, Stoke-on-Trent. |
| Halifax Fever Hospital; £12. | Southport Children's Sanatorium; £9. |
| Lancashire Infirmary. | Tamworth Cottage Hospital. |
| Leeds Nurses' Institution. | |

### Amusements and Relaxation.

### N.B.—New Word Competition Commences April 5th, 1890, ends June 28th, 1890.

Three prizes of 15s., 10s., 5s., will be given for the largest number of words derived from the words set for dissection.

Proper names, abbreviations, foreign words, words of less than four letters, and repetitions are barred; plurals, and past and present participles of verbs, are allowed. Nuttall's *Standard* dictionary only to be used.

N.B.—Word dissections must be sent in WEEKLY, arranged alphabetically, with correct total affixed.

The word for dissection for this, the FIRST week of the quarter, being

"EASTER."

| Names. | Mar. 27. | Totals. |
|---|---|---|
| Holland | 63 | 1055 |
| Red Cape | — | 132 |
| Nurse Marie | — | 499 |
| Garfield | — | 243 |
| Midget | 57 | 894 |
| E. M. S. | — | 149 |
| Sister | — | 591 |
| Duchess | 52 | 637 |
| Ellice | 64 | 1076 |
| Reldas | 66 | 1115 |
| Jenny Wren | 60 | 971 |
| Mopus | — | 122 |
| Neptune | — | 118 |
| Qu'appelle | 57 | 996 |
| Whitehoe | — | 880 |
| Nurse Clague | — | 40 |
| Lauriston | | 453 |
| B. E. T. | — | 110 |
| Jacko | — | 258 |
| Lady Clara | — | 1109 |
| Patience | 63 | 1091 |
| Ecila | 60 | 690 |
| Maude | — | 698 |
| Ruby | — | 1088 |
| Esperance | 68 | 1089 |
| M. W. | 68 | 698 |
| Crystal Palace | — | 755 |
| Jackanapes | — | 25 |
| M. M. M. | — | 318 |
| A. F. | — | 49 |
| E. S. | — | |

Results of Fourth Quarterly Word Competition will be published next week.

### Notice to Correspondents.

N.B.—All letters referring to this page which do not arrive at 140, **Strand, London, W.C.**, by the *first post on Thursdays*, and are not addressed PRIZE EDITOR, will in future be disqualified and disregarded.

